# Postural correlates with painful situations

**DOI:** 10.3389/fnhum.2013.00004

**Published:** 2013-02-05

**Authors:** Thierry Lelard, Benoît Montalan, Maria F. Morel, Pierre Krystkowiak, Said Ahmaidi, Olivier Godefroy, Harold Mouras

**Affiliations:** ^1^EA 4559, Laboratoire de Neurosciences Fonctionnelles et Pathologies, UFR de Médecine, Université de Picardie Jules VerneAmiens, France; ^2^Structure Fédérative de Recherche en Santé CAP-Santé, Université de Picardie Jules Verne, Amiens and Université de Reims-Champagne-ArdennesReims, France; ^3^Laboratoire ICONES (EA4699), Normandie Université, U.F.R. des Sciences de l'Homme et de la Société76821 Mont-Saint-Aignan Cedex, France; ^4^Service de Neurologie, CHU AmiensAmiens, France; ^5^EA 3300, Adaptations Physiologiques à l'Exercice et Réadaptation a l'Effort, UFR des Sciences du Sport, Université de Picardie Jules VerneAmiens, France

**Keywords:** empathy for pain, posturography, embodiment, socioaffective neuroscience, affiliation

## Abstract

**Background:** Emotional context may play a crucial role in movement production. According to simulation theories, emotional states affect motor systems. The aim of this study was to compare postural responses assessed by posturography and electromyography when subjects were instructed to imagine themselves in a painful or a non-painful situation.

**Methods:** Twenty-nine subjects (22.3 ± 3.7 years) participated in this study. While standing quietly on a posturographic platform, they were instructed to imagine themselves in a painful or non-painful situation. Displacement of the center of pressure (COP), leg muscle electromyographic activity, heart rate, and electrodermal activity were assessed in response to painful and non-painful situations.

**Results:** The anteroposterior path was shorter (*p* < 0.05) when subjects imagined themselves in a painful situation (M = 148.0 ± 33.4 mm) compared to a non-painful situation (158.2 ± 38.7 mm). Higher tibialis anterior (TA) activity (RMS-TA = 3.38 ± 1.95% *vs.* 3.24 ± 1.85%; *p* < 0.001) and higher variability of soleus (SO) activity (variation coefficient of RMS-SO = 13.5 ± 16.2% *vs.* M = 9.0 ± 7.2%; *p* < 0.05) were also observed in painful compared to non-painful situations. No significant changes were observed for other physiological data.

**Conclusion:** This study demonstrates that simulation of painful situations induces changes in postural control and leg muscle activation compared to non-painful situations, as increased stiffness was demonstrated in response to aversive pictures in accordance with previous results.

## Introduction

The interrelation between the motor and affective components of behavior has been studied for a long time. For example, one of the first attempts to study the human mind was conducted by Plato using one of his philosophical models, the tripartite structure of the soul, which had a profound influence on psychology research. As noted by Popper (1968), “Plato's structure of the soul is characterized by an unstable equilibrium—indeed a schism—between its upper functions, the instincts or appetites.” As part of this history, Charles Darwin also made a major contribution by arguing that an emotion induces adaptation of behavioral responses according to the environmental context that triggered this emotion (Darwin, 1872). Thus, the automatic responses triggered by emotional stimuli play a central role for survival of the species and reproduction (Campbell et al., 1997) and can be viewed as instinctual responses (Panksepp and Biven, 2012).

Some studies suggest that emotions influence motor processes (Michalak et al., 2009; Schmidt et al., 2009; Naugle et al., 2011; Coombes et al., 2012). Several authors have tried to explain behavior by means of a biphasic model in which emotional stimuli should be considered as appetitive or defensive (Lang et al., 2008) and might result in approach-withdrawal responses. The corresponding hypothesis is that emotion shapes behavior so that pleasant events should trigger approach whereas unpleasant events should trigger withdrawal.

This interrelation between behavior and emotion is also supported by neuroanatomical data regarding the interface between limbic and motor neural circuits. For example, the basal ganglia are involved in involuntary movements (such as gait and posture), but also in the physiological expression of emotions (Kandel et al., 2000).

Posturography determines displacement of the center of pressure (COP) and is appropriate to demonstrate postural changes and quantify body movements accompanying approach-withdrawal behaviors (Gurfinkel, 1973; Winter et al., 1990). In recent studies, this method was used to record motor responses-induced by emotional stimuli while subjects remained in bipedal and/or unipedal stance. Presentation of emotional pictures [International Affective Picture System (IAPS); Lang et al., 2008] has been shown to induce an approach-withdrawal behavior (Hillman et al., 2004) or freezing responses (Hillman et al., 2004; Azevedo et al., 2005; Facchinetti et al., 2006; Stins and Beek, 2007). These postural responses were recorded in response to negative stimuli such as disgusting aversive pictures depicting mutilation and can be defined as “instinctual responses.”

Pain includes a subjective experience triggered by activation of a mental/neural representation of actual or potential tissue damage supporting the affective component of pain and inducing aversion that motivates termination or reduction of behavior, or induces escape behavior to avoid exposure to the noxious stimulation (Price, 2000).

Simulation of another subject's behavior or imagination of a visual situation experienced by ourselves involve simulation processes and activation of internal models (Zahavi, 2008). The ability to simulate a situation explains the mechanism by which we can understand another person's actions and the induction of the bodily expression of emotion. Simulation of one's own behavior is based on the ability of an individual to simulate actions, to simulate perception and to anticipate (Hesslow, 2002, 2012). During simulation processes, the subject may replay her own past experience in order to extract from it pleasurable, motivational, or strictly informational properties (Dokic and Proust, 2002). According to the embodiment theories, experiencing emotional states affects motor systems (Giummarra et al., 2008; Michalak et al., 2009; Kiefer and Pulvermuller, 2012). Simulation of a situation is supported by the discovery of mirror neurons (Gallese et al., 1996; Rizzolatti et al., 1996; Thioux and Keysers, 2010), responding both during action production and observation of the same action performed by another person. Hutchison et al. (1999) have shown that there are pain-related neurons in the anterior cingulate cortex (ACC) that respond both to thermal stimulation and also to the observation of the same thermal stimulation delivered to another individual (Hutchison et al., 1999).

An individual who is imagining a situation involve representational characters rather than instinctual characters (Giummarra et al., 2008; Michalak et al., 2009; Kiefer and Pulvermuller, 2012). For instinctual characters, emotional propensities arise out of subcortical structures and activate quite automatic-visceral and bodily outputs. On the other hand, for representational characters, emotional propensities arise out of cortical structures. For example, similar fronto-parietal network is activated in pianist participants when they played music and when they imagined playing the same music (Meister et al., 2004).

According to the perception-action model (Preston and De Waal, 2002), empathy activates somatic and autonomic responses. Simulation of a painful situation may therefore be an efficient functional context involving emotional information processing associated with the promotion of protective or recovery visceromotor and behavioral responses. The ability to experience the emotion observed in others implies a physiological synchrony between the observer and the observed individual (Levenson and Ruef, 1992). The automatic coupling mechanism between perception and action would be used to predict and understand the other person's behavior (Rizzolatti et al., 2001). This ability to simulate another person's emotional response in a particular situation could be the basis for the development of empathic skills (Meltzoff and Decety, 2003). The instruction to adopt another person's perspective modulates pain rating according to the affective link between the observer and the individual experiencing the outcome (Singer et al., 2006; Penner et al., 2008). To address the question of whether motor response is modulated by perspective taking, it must be determined whether differential motor responses are observed when viewing pictures depicting painful situations compared to non-painful situations.

The aim of this study was therefore to record differential postural responses as measured by posturography and electromyography when subjects were instructed to imagine themselves in a painful or non-painful situation within the functional context of empathy for pain.

Visual pain stimuli and instructions to embody the displayed situation were hypothesized to induce postural adaptation variations that could be quantified by changes in the trajectory of the body's COP. Considering the inverted pendulum model, in quiet standing, an ankle strategy applies in the antero-posterior direction (Winter et al., 1990). Leg muscles activation (tibialis anterior and soleus) reflect forward or backward leaning of the whole body. Carpenter et al. (2001) also reported changes in TA or SOL activation during a postural threat condition that was attributed to a freezing response (Carpenter et al., 2001).

## Materials and methods

### Participants

Thirty participants (13 males; mean age and SD = 22.3 ± 3.7) were included with (1) no history of visual or motor impairment, (2) no prior or current treatment for psychiatric or neurological disorders. All participants signed an informed consent form. The experimental procedures were in accordance with the ethical standards of the Helsinki declaration and were approved by the local ethics committee (CPP Nord Ouest 2).

### Stimulus materials

Ten pictures depicting painful or non-painful situations involving the hands or feet were selected from a larger database validated in previous studies (e.g., Jackson et al., 2005). Participants were instructed to imagine that they had experienced the situations that they were about to see. Stimuli presentation was controlled by a computer running E-Prime software (Psychology Software Tools, Inc., Pittsburgh, PA, USA).

### Posturography and physiological data assessments

Posturography and physiological data were recorded using a Biopac MP150 system (Biopac Inc., Santa Barbara, CA). Movements of the COP were recorded during the rest stance by a posturographic platform (Satel, Blagnac, France). Analogue data from three strain gauges were recorded and movements of the COP in the anteroposterior (AP) and mediolateral (ML) directions were computed by AcqKnowlege software (Biopac Inc., Santa Barbara, CA).

Electromyography (EMG), electrocardiography (ECG), and electrodermal activity (EDA) were recorded at a rate of 1000 Hz by a MP-150 Biopac System. Heart rate (HR) was recorded with a standard Lead-II electrocardiogram using three disposable electrodes (EL503). EDA was recorded with two Ag/AgCl electrodes (GSR100C, Biopac Inc., Santa Barbara, CA) filled with an isotonic paste attached to the volar surface of the index and middle fingers of the subject's hand. A constant-voltage device was used to pass 0.5 V between electrodes. EMG activity of leg muscles was recorded with disposable electrodes (EL503). The electrodes were fixed (2 cm apart center to center) over the tibialis anterior (TA) and soleus (S) muscle bellies.

Respiratory activity was recorded via a transducer (TSD201) recording chest circumference variations.

### Procedure

Firstly, participants stood barefoot in the middle of the force plate. They were asked to maintain a comfortable bipedal stance with their arms hanging relaxed alongside their body and their feet pointing 30° outward. Visual stimuli were then presented 2 m in front of the participants using a video projector. Participants were instructed to watch the images presented without any additional movement and to imagine the pain that they would experience in the situations displayed. Pictures of painful and non-painful situations were presented in random order. For each picture, a trigger corresponding to each type of emotional stimulus was sent to the Biopac MP150. During a first recording session, 5 images were presented for 12 s. In order to avoid tiredness, participants were asked to stretch their legs or sit according to their preference. During a second session, 5 images were presented for the same duration. For each trial, stimulus presentation was preceded by a fixation cross for 0.5 s. The stimulus was then presented for 12 s with an inter-stimulus interval of 2 s.

Secondly, participants performed a pain judgment task of the painful and non-painful pictures (Jackson et al., 2005). At the beginning of the acquisition sequence, the participants were instructed to imagine the pain they would experience in the situations displayed. The trial sequence started with a fixation cross for 0.5 s. The stimulus was then presented until the participant's response. After responses, an inter-stimulus interval of 1 s was added. Immediately after onset of the stimulus, subjects were instructed to indicate their ratings by using their right hand to press 1 of 9 computer keys (with scores ranging from 0 = no pain to 9 = very severe pain).

### Data analysis

The mean postural response to both painful and non-painful situations was calculated for each experimental condition. The following indices were calculated for each trial: (1) the mean COP position in the anteroposterior direction (COP-AP), reflecting the extent to which a participant leaned toward the anterior or posterior direction during a 12-s trial; (2) the length of the sway path of the COP in the anteroposterior direction (Length [COP]-AP), reflecting the degree of body sway in the AP direction; (3) the area encompassed by displacements of the COP (COP-Area), corresponding to the surface of the confidence ellipse containing 90% of the sampled COP positions.

The level of muscle activation was quantified by calculating the root mean square (RMS) of raw data over 0.5 s with a sliding window. RMS-TA and RMS-SO indicated the level of activation of TA and SO muscles, respectively. Var-TA and Var-SO indicated the variation of TA and SO muscles (SD/mean), respectively. Heart rate (HR) was calculated from ECG data by AcqKnowledge software.

### Statistical analysis

Postural, physiological and pain rating data were submitted to a paired samples *t*-test to compare the response during painful and non-painful situations. Pearson's correlation coefficients between the subjects' rating in the pain judgment task, the posturographic parameters and the physiological responses were also calculated. A *p* < 0.05 value was considered statistically significant.

## Results

### Pain judgement task

**Figure 1:**
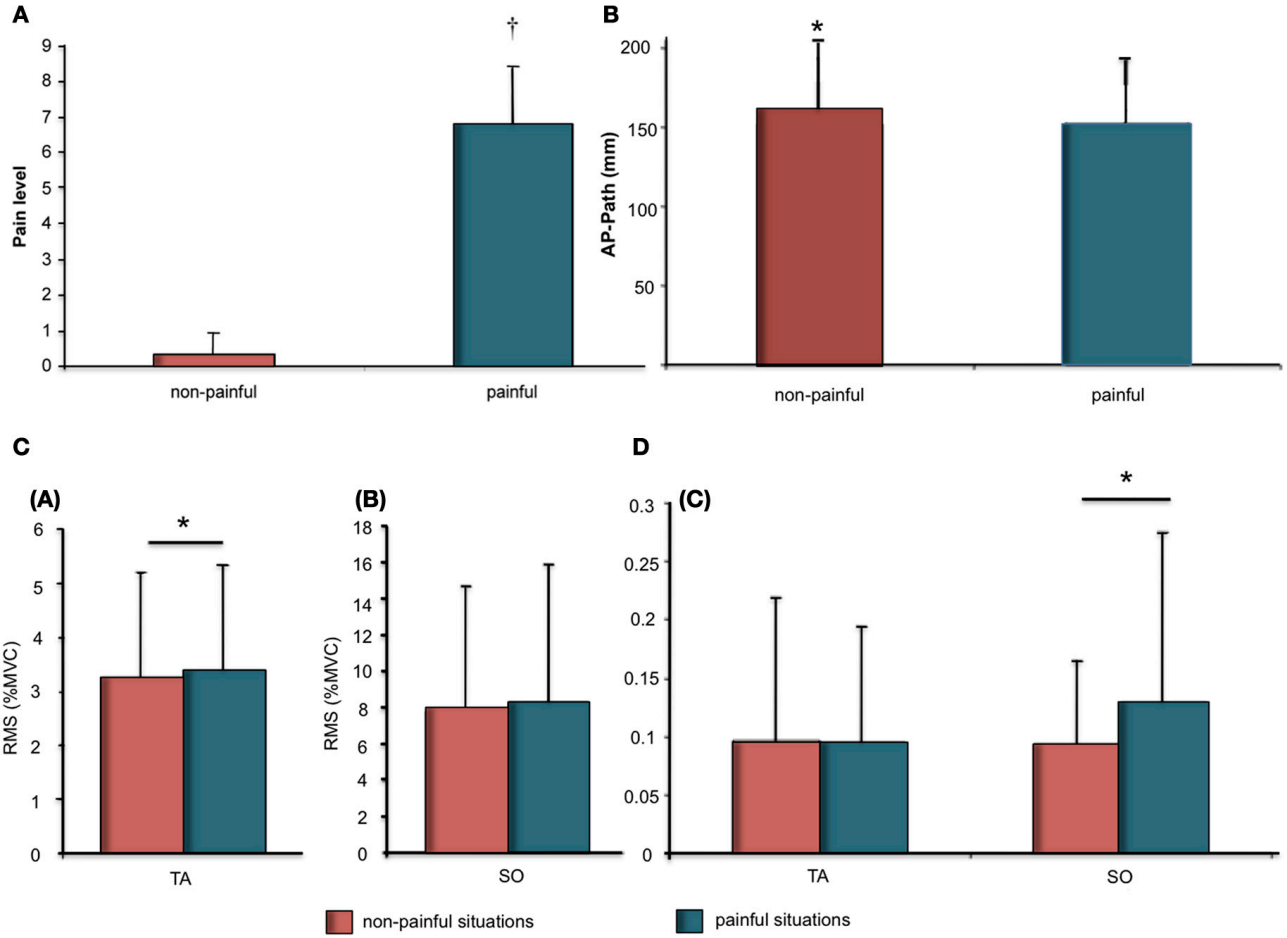
**(A)** Pain ratings (Mean and SD) as a function of stimuli (Painful vs. Non-painful). Significant differences are indicated as: ^†^*p* < 0.001; **(B)** Means and SDs of the anteroposterior length as a function of stimuli (painful vs. non-painful); **(C)** Means and SDs of electromyographic data for Soleus (SO) and Tibialis Anterior (TA) muscles. **(a)** RMS as % of MVC for TA as a function of stimuli (painful vs. non-painful). **(b)** RMS as % of MVC for SO as a function of stimuli (painful vs. non-painful); **(D) (c)** Variability of RMS as a function of stimuli (painful vs. non-painful). Significant differences are indicated as: ^*^ *p* < 0.05.

The *t*-test revealed a significant difference in mean pain ratings for painful stimuli (*M* = 6.80 ± 1.64) compared to non-painful stimuli (*M* = 0.33 ± 0.60) (Figure 1A).

### Postural responses to visual stimuli

COP displacement during the 12-s presentation were demonstrated in response to painful as compared to non-painful stimuli (Figure 1B). The *t*-test revealed a significant effect for stimuli on AP path (*t* = −2.34; *p* < 0.05). AP path was shorter during presentation of painful visual stimuli (*M* = 152.0 ± 41.7 mm) compared to non-painful visual stimuli (160.7 ± 43.2 mm).

### Physiological responses to visual stimulation

Physiological responses were recorded during the 12-s presentation of painful or non-painful stimuli (Figures 1C,D; Table 1). The *t*-test revealed a higher RMS-TA (*t* = 2.20, *p* < 0.05) when subjects imagined themselves in a painful situation (*M* = 3.38 ± 1.95% AU) compared to a non-painful situation (*M* = 3.24 ± 1.85%). No significant differences for var-TA were observed between painful (*M* = 9.53 ± 10.05%) and non-painful situations (*M* = 9.64 ± 12.43%). RMS-SO was also not significantly different between painful (*M* = 8.31 ± 6.92%) and non-painful situations (*M* = 7.72 ± 7.63%). *T*-test revealed a higher var-SO value during painful stimuli (*M* = 12.83 ± 14.96%) compared to non-painful stimuli (*M* = 9.36 ± 7.24%, *t* = 2.62, *p* < 0.05).

**Table 1:** **COP displacement and physiological changes as a function of stimuli (painful vs. non-painful)**.

	**Painful situation mean and (SD)**	**Non-painful situation mean and (SD)**
COP-AP (mm)	0.69 (2.40)	0.01 (1.57)
COP-Area (mm^2^)	172.30 (242.98)	153.97 (170.00)
Length [COP]-AP (mm)	151.99 (42.42)	160.75 (43.97)^*^
RMS-TA (%)	3.38 (1.95)	3.24 (1.85)
Var-TA (%)	9.53 (10.05)	9.64 (12.43)
RMS-SO (%)	8.31 (6.92)	7.72 (7.63)
Var-SO (%)	12.83 (14.96)	9.36 (7.24)^*^
HR (bpm)	94.55 (14.93)	94.11 (14.60)
EDA (AU)	150.93 (90.42)	150.08 (90.62)

Significant differences are indicated as:

* p < 0.05.

No significant differences for heart rate and electrodermal activity were observed between painful and non-painful stimuli (Table 1).

Correlations between postural, physiological data, and pain judgment were computed (Table 2). Pain rating was correlated with Length [COP]-AP (*r* = −0.22). Physiologic data were also correlated with posturographic parameters, heart rate was correlated with COP-Area (*r* = 0.22) and Length [COP]-AP (*r* = 0.38).

**Table 2:** **Correlation between postural data, physiological data, and pain rating**.

	**HR**	**EDA**	**Pain rating**
COP-AP	0.053	−0.084	0.001
COP-Area	0.218^*^	−0.136	−0.076
Length [COP]-AP	0.388^**^	−0.167	−0.220^*^
HR		0.063	0.058
EDA			−0.019

Significant differences are indicated as:

* p < 0.05;

** p < 0.01.

## Discussion

This study investigated postural changes and physiological correlates-induced by pictures depicting painful and non-painful situations of daily living. We hypothesized that simulation of painful situations would induce a motor response characterized by changes in COP trajectory. This study confirmed that a 12 s presentation of emotionally charged stimuli-induced postural and physiological responses.

Presentation of emotional pictures has already been shown to affect equilibrium. However, to our knowledge, with the present study this is the first time that the experimental set up favors more representational and cognitively loaded emotions than instinctual responses to study whole body movement. Indeed, postural responses were obtained while subjects were instructed to imagine themselves in the painful and non-painful situations. The link between pain rating and body placement was found for the Length [COP]-AP. Firstly, postural changes were observed in the AP direction in response to the painful situation, confirming previous data recorded when subjects viewed pictures of mutilations (Azevedo et al., 2005; Facchinetti et al., 2006; Stins and Beek, 2007). Indeed, COP displacements were reduced during presentation of painful stimuli. We also described a negative correlation between length of COP-AP and pain rating, confirming the appearance of stiffening response to pain visual simulation. For visual stimuli rated the most painful, simulation induces a decrease of length of COP-AP. This point brings some evidence for a representational interpretation of the present study. Indeed, postural responses seem to be dependent of the perceived pain during simulation. Our results therefore confirmed that 12 s presentation of painful situations (Jackson et al., 2005) combined with instructions to imagine oneself in the situation displayed-induced postural modulations in the participants. According to previous studies, the reduction of COP excursion-induced by negative stimuli was explained by adoption of a freezing strategy (Hillman et al., 2004; Azevedo et al., 2005; Facchinetti et al., 2006; Stins and Beek, 2007). Many of these studies used aversive pictures from IAPS with high arousal, causing a feeling of disgust. The AP trajectory of the COP describes persistent adaptation of posture in response to a 12-s presentation of a painful situation, whereas backward motion of the COP might be identified with a shorter latency.

Secondly, the postural adjustments observed during the 12-s presentation of pain stimuli were also accompanied by physiological changes. We also described some links between length of COP-AP, COP-Area, and HR during picture presentation. Decrease in HR was associated with decrease in COP-Area and length of COP-AP. Changes in postural muscle activity in response to emotional stimuli were also described. The increase of RMS-TA, characterizing TA muscle tone, reflects adoption of a stiffening strategy. General stiffness has been reported in response to anxiety (Fridlund et al., 1986). A similar increase in RMS-TA has been previously associated with increased anxiety caused by a postural threat (Carpenter et al., 2001). Participants also exhibited an increased Var-SO in response to painful situation. Var-SO represents the coefficient of variation of muscle activity and may be related to increased postural adjustments or a motor response to the stimuli. To our knowledge, this is the first study to record postural responses simultaneously with changes in postural muscle activity. Up until now, EMG data recorded during presentation of emotional faces have been used to describe changes in facial muscle activity and mimicry (Sonnby-Borgstrom, 2002; Balconi et al., 2011). Several studies have demonstrated that EDA increases with the arousal-induced by visual stimuli (Lang et al., 1993; Horslen and Carpenter, 2011) and changes in heart rate have been previously described with freezing responses (Azevedo et al., 2005; Facchinetti et al., 2006; Stins and Beek, 2007). Our results are not consistent with these previous studies showing changes in physiological responses to aversive stimuli, probably because the painful pictures used in this study (Jackson et al., 2005) may have had a lower arousal level than the mutilation pictures used in previous studies. This difference should also be explain by the representational character of the present task whereas previous studies describe instinctual response to visual stimuli. However, changes in postural activity and muscle activation demonstrate the effect of simulation of painful situations. This response could also be explained by the involvement of a cognitive process in mental simulation. Mental simulation represents the cognitive process by which we can mentally represent perceptual information in the absence of appropriate sensory input (Munzert et al., 2009). This mental simulation is based on internal simulation of actions (Jeannerod, 2001; Grush, 2004). In order to simulate these scenes, the subject must be able to understand whether or not the scene describes a painful or non-painful situation. Moreover, evaluation of the valence of a stimulus occurs immediately and without attention leading to an automatic response (Bargh et al., 1996; Eerland et al., 2012). Moreover, a previous study reported that pictures representing attacks and pictures of human mutilation prompted the greatest evidence of defensive activation (Bradley et al., 2001). The contents of aversive stimuli are the most threatening from a survival perspective and responses to aversive stimuli are reflex responses that have evolved to facilitate survival of individuals and species (Rolls, 2000; Bradley et al., 2001; Lang and Bradley, 2010).

Further investigation should be conducted to identify the differences between responses to aversive stimuli (viewing aversive visual stimuli) and simulation of a painful situation. The present study demonstrate correlation between some variables (posturographic parameters, physiological parameters and pain rating). However, to confirm the correlation between pain rating and the other parameters, further works should use several categories of the perceived intensity of pain (not limited to low or high as in the present study).

## Conclusion

This study highlights the relationship between simulation of painful situations and postural modulation and physiological responses (leg muscle activation). Changes in postural muscle activity and COP displacement during simulation of a painful situation were also consistent with adoption of a freezing strategy during the 12-s presentation of the stimuli.

Modulation of postural responses during painful simulation lays the basis for further studies concerning the role of perspective-taking in motivational dimension of motor control and social interaction. The present results using representational stimuli (imagining themselves experiencing pain) show similar results with previous study using instinctual stimuli (viewing negative stimuli). However, several studies are carried out to understand the mechanisms underlying motor responses during complex representational processes such as empathy. The effects of embodiment of painful situation should also be studied in further work by comparison of both conditions (viewing vs. imagining painful situation).

### Conflict of interest statement

The authors declare that the research was conducted in the absence of any commercial or financial relationships that could be construed as a potential conflict of interest.
